# Hepatocellular carcinoma: preclinical data on a dual-lumen catheter kit for fibrin sealant infusion following loco-regional treatments

**DOI:** 10.1186/1750-9378-9-39

**Published:** 2014-11-24

**Authors:** Francesco Izzo, Vittorio Albino, Raffaele Palaia, Mauro Piccirillo, Fabiana Tatangelo, Vincenza Granata, Antonella Petrillo, Secondo Lastoria

**Affiliations:** Istituto Nazionale per lo Studio e la Cura dei Tumori di Napoli, Fondazione “G. Pascale”, Via M.Semmola, 80131 Naples, Italy

**Keywords:** Hepatocarcinoma, Dual lumen catheter, Sealant, Loco regional treatments

## Abstract

**Background:**

Fibrin sealants are currently used in a variety of surgical and endoscopic settings to improve time to haemostasis, reduce blood loss and complications. However, the application of sealants (composed of two essential components: fibrinogen and thrombin) is not without difficulties. These sealants are normally applied to the resected area using dual-chamber delivery systems. Administration of these substances with different viscosities and diverse flow rates through a long catheter means that a certain amount of force needs to be applied and clot formation and clogging at the distal end of the catheter can occur.

**Methods:**

We designed a novel dual-lumen catheter to facilitate the optimal application of fibrin sealant after diagnostic and therapeutic percutaneous procedures and assessed the efficacy and tolerability of this dual-lumen kit when used in a model of hepatic fine needle aspiration (FNA) biopsy and radiofrequency ablation (RFA) in an *in vivo*, preclinical porcine study.

**Results:**

The experimental was performed on nine pigs (mean body weight 85 ± 7 kg) and with the exception of one pig, all animals survived in good conditions until the day of hepatectomy and euthanasia. The premature death of this animal was in the veterinarian’s judgment caused by a common, non-infective disease. In all nine pigs, bleeding was stopped within 3 minutes of the application of the fibrin sealant and no cases of recurrent bleeding occurred.

**Conclusions:**

The new dual aspect catheter increased ease of delivery of the sealant and FNA liver biopsy and RFA procedures were successfully and safely performed.

## Background

Hepatocellular carcinoma (HCC) is one of the most common human solid malignancies worldwide [1, 2]. The liver is also a frequent site of metastases from non-hepatic malignancies [2]. Surgical resection of the tumour is an effective treatment. However, less than 10–30% of primary and secondary hepatic malignancies are resectable due to tumour number/location, and the risk of liver failure after partial hepatectomy [3, 4]. A number of cytodestructive treatments are available for patients with unresectable hepatic tumours including thermal ablation techniques such as radiofrequency ablation (RFA) which has been increasingly used in recent years [5, 6]. RFA is not without complications, which range from 6.3–9.5% according to current estimates [2, 7–9]. Frequently reported complications associated with percutaneous RFA include abdominal haemorrhage, bile leakage, biloma formation, hepatic abscesses, and neoplastic seeding [2, 7–9]. Abdominal bleeding and neoplastic seeding are also associated with percutaneous diagnostic hepatic procedures as highlighted in a recent review involving patients who underwent fine needle aspiration (FNA) liver biopsy [10].

Control of intraoperative haemorrhage is particularly difficult when the site is poorly accessible, in the presence of altered coagulation parameters and in patients with congenital or acquired bleeding disorders. Parenchymal organs, such as the liver, pose additional problems due to their soft nature and propensity to bleed. Several techniques have been developed over the past decades to ensure haemostasis during liver resection, including selective suture, electrocautery, argon beam coagulation, and various manoeuvers [11, 12]. Haemostatic agents for topical application have also been developed as additional measures to promote haemostasis and tissue sealing [13]. Among these, fibrin sealants also referred to as fibrin glues, have been used in a variety of surgical and endoscopic settings [14–16]. Fibrin sealants are composed of two essential components, fibrinogen and thrombin, plus other ingredients including factor XIII (for fibrin cross-linking), calcium chloride and aprotinin (for prevention of clot fibrinolysis) [17]. Fibrin sealants promote haemostasis by mimicking the final stage of the blood coagulation cascade. Clot formation is therefore possible independently of the patient’s coagulation status or antithrombotic medication.

Fibrin sealant preparations are currently commercially available and are successfully used in clinical practice worldwide. However certain limitations in application techniques have been observed. They are normally applied to the resected area using dual-chamber delivery systems, with one chamber containing fibrinogen and the other thrombin. Application of fibrin sealants in open surgery is relatively straight-forward [17], but delivery through an injection catheter in endoscopic procedures is more problematic as considerable force may be required to inject the highly viscous fibrinogen component through a long catheter [14, 18, 19]. In addition, because the two sealant components have different viscosities and consequently diverse flow rates through the catheter, inappropriate clot formation at the distal end of the catheter and clogging can occur.

We describe here a novel type of dual-lumen catheter designed to facilitate the optimal application of fibrin sealant after diagnostic and therapeutic percutaneous procedures. The main objectives of this *in vivo,* preclinical study were to determine the ease-of-use and/or limitations of this new catheter and to test the efficacy and safety of the haemostatic treatment when applied via the dual-lumen catheter in a porcine model of hepatic FNA biopsy and RFA.

## Materials and methods

### Catheter design

The dual-lumen catheter was designed for the application of Tisseel® (Baxter) and other fibrin sealants following percutaneous liver procedures including FNA biopsy and RFA. Fibrinogen and thrombin solutions have different viscosities and therefore different flow rates through the catheter lumen which mean they are not delivered simultaneously and in equal amounts to the injury site leading to inadequate sealant application and clot formation. The aim was to design a catheter through which the two components would flow at similar rates and would be delivered simultaneously and in equal amounts at the site of injury thus reducing the time and force necessary to inject the sealant.

The first step was to modify the lumen of the catheter based on the density and viscosity of each solution. Using Poiseuille’s law it established that the optimal ratio between the surface of the lumen cross section containing the fibrinogen solution (the more viscous solution) and that of the lumen containing the thrombin solution (less viscous) should be greater than the square root of the ratio of the viscosities of both solutions. The dual-lumen catheter of in the present study was then manufactured based on these dimensions.

### Kit and procedure for catheter use

**Figure 1:**
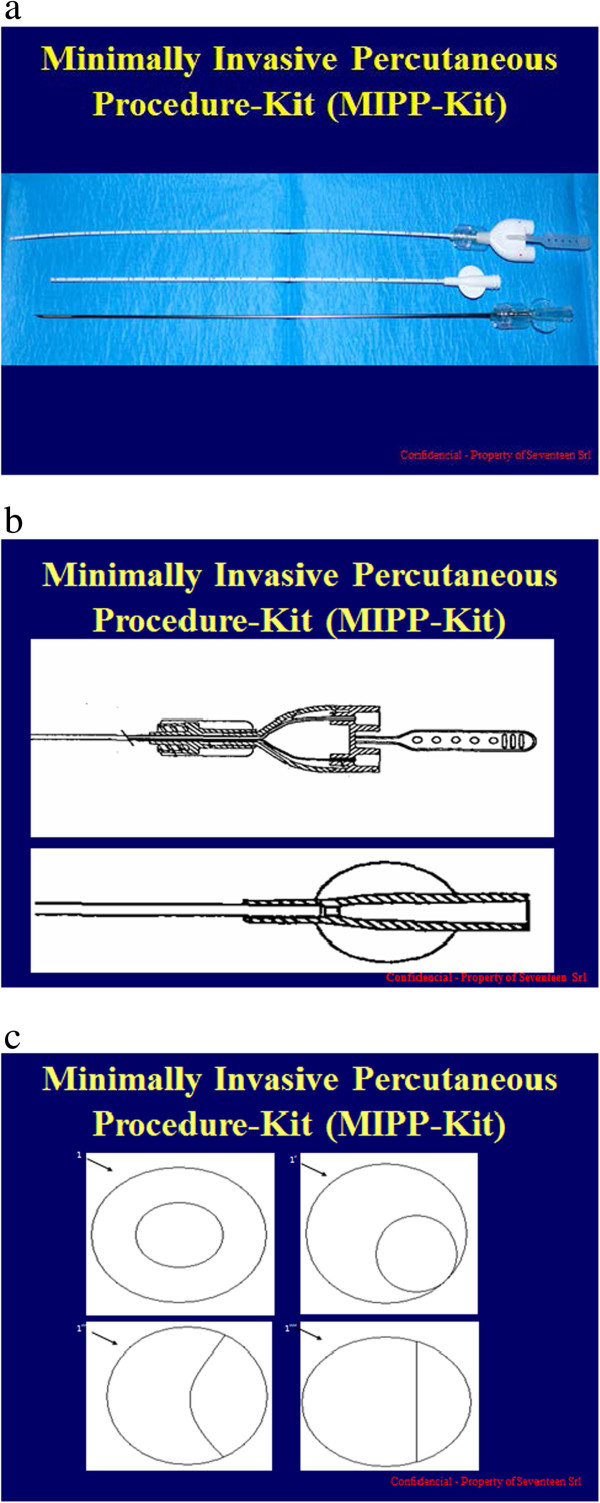
**Design of the dual-lumen catheter. (a)** introducer (14G), coaxial dual-lumen catheter (15G) and needle **(b)** longitudinal section showing introducer, catheter and needle **(c)** transverse section showing diameters of lumen.

**Figure 2:**
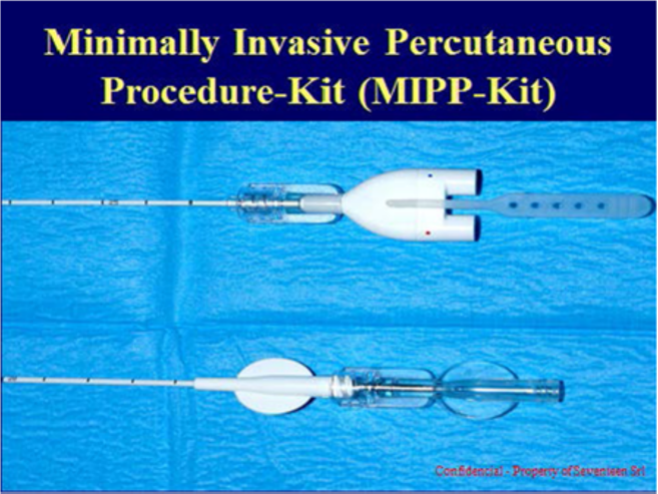
**Patented minimally invasive percutaneous procedure-kit (MIPP-Kit).**

**Figure 3:**
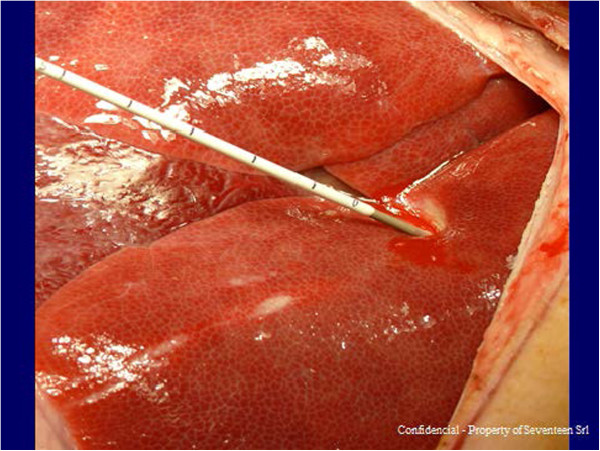
**Needle being inserted in liver parenchyma.**

The kit used in the present study consisted of a 14G introducer, a 15G coaxial dual-lumen catheter, and a needle (Figure 1a,b,c). The introducer (Grimalind® L25) and catheter (20 cm long) were graduated, of the same length (200 mm) and made of radiopaque material (30% barium sulfate). The catheter had a steel core, a sharp and removable tip that was slightly longer than the introducer (210 mm) and a Luer-Lock connection. The needle had an oblique tip to allow easy penetration of the tissue. The catheter composed of Grilfex® ELG 6260 (PEBA) — a radiopaque and semi-rigid material — has two chambers one for each sealant component and has now been granted and an Italian patent (0001395842) and an international patent is pending (PCT/IT2010/000241) (Figure 2). To perform a biopsy/percutaneous therapeutic procedure, the appropriate introducer is positioned using US, CT or MR imaging guidance (Figure 3). After removing the core of the introducer, the appropriate needle for the procedure is inserted and when treatment is completed the needle is removed and the coaxial, dual-lumen catheter for sealant application is inserted. The sealant is injected into the biopsied or ablated area, along the introducer track, while the introducer is carefully removed.

### Animals and treatments

The experimental procedure was performed on Large White pigs. Each animal received an identification number and data on each animal were recorded in a database, under the supervision of the principal investigator. The health status of the animals, including the results of laboratory tests, was assessed by a veterinary. The study was approved by the local Ethics Committee for animal experimentation.

**Figure 4:**
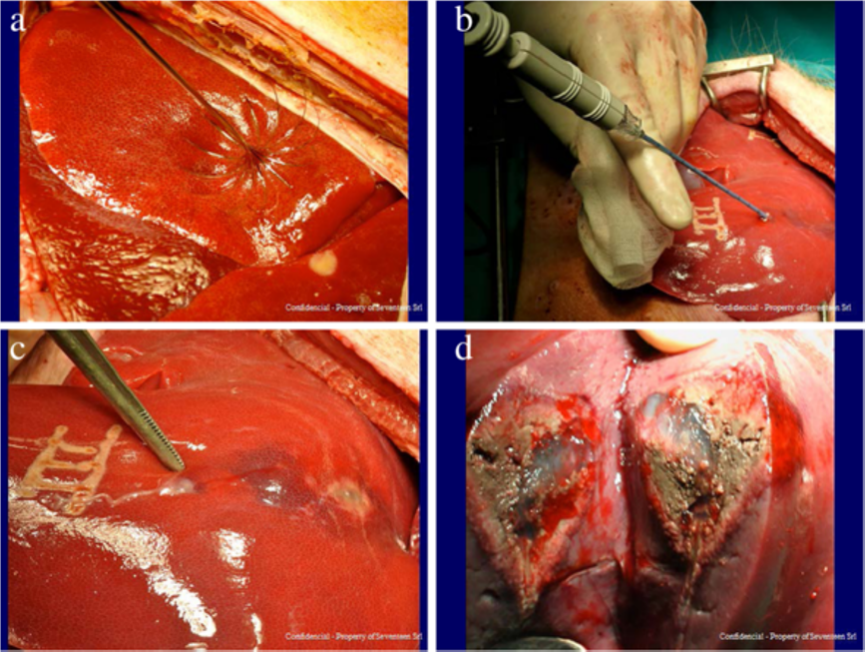
**Radiofrequency ablation procedure: (a) radio frequency electrode, (b) electrode inserted in the liver parenchymal, (c) surface of the liver parenchyma where electrode was inserted, (d) section of the liver parenchymal showing necrosis and Tisseel.**

The pigs were randomly assigned to three groups, based on the length of follow-up after the intervention. Pigs in Group 1 had no follow-up (sub-acute outcome), pigs in Group 2 were followed for 2 weeks, (2-week outcome) and pigs in Group 3 for 4 weeks (4-week outcome). All animals underwent laparoscopic examination under general anaesthesia. Three liver FNAs with 18G needle from one liver lobe and three RFA with 14G needle electrode, from Boston Scientific (maximum 250 watts) at three distinct liver lobes were performed on each animal (Figure 4). After assessment of bleeding severity, Tisseel® (10 ml) (Baxter) was applied via the novel catheter in the treated areas and along the path of the needle used (Figure 4). Pigs in Group 1 underwent total hepatectomy and were euthanized 24 hours after the intervention, while pigs in Groups 2 and 3 were hepatectomized and euthanized 2 and 4 weeks after the intervention, respectively.

### Assessments

The primary efficacy measure was achievement of haemostasis within 3 minutes of application of fibrin sealant. If bleeding persisted after 3 minutes the surgeon was free to use any other technique of his/her preference to control the haemorrhage. At the end of the procedure but before suture, sites treated with fibrin sealant were checked again to make sure they were not bleeding. Throughout the procedure all bleeding events were recorded as were any complications and side-effects described as mild/moderate or severe, and as treatment related/unrelated to treatment according to the investigator’s judgment.

Coagulation parameters were monitored before surgery, before and 24 hours after anaesthesia and at the end of the follow-up before total hepatectomy. Blood analyses were carried out on all animals in Groups 2 and 3 each week until total hepatectomy. Removed livers were assessed by macroscopic and microscopic analysis to detect any changes caused by FNA, RFA and fibrin sealant application. All specimens were stained with haematoxylin and eosin, and for nicotinamide adenine dinucleotide (NADH) diaphorase activity.

## Results

The experimental procedure was performed on nine pigs (3 pigs per Group) with a mean body weight of 85 ± 7 kg. With the exception of one pig in Group 3, all animals survived in good conditions until the day of hepatectomy and euthanasia. The premature death of this animal was in the veterinarian’s judgment caused by a common, non-infective disease. In all nine pigs, bleeding was stopped within 3 minutes of the application of the fibrin sealant and no cases of recurrent bleeding were observed. The procedure was completed in all cases and surgical techniques to achieve haemostasis were not required. The interventions (FNA and RFA) were not associated with complications as anticipated including biloma, hepatic abscess, and bile duct injury. The absence of these complications typically associated with the two interventions, was confirmed by anatomical and pathological assessments of the explanted livers. Blood tests did not reveal any abnormal coagulation parameters. The kit was easy to use and the application of the fibrin sealant did not pose any particular technical problems. The fibrin sealant could be applied correctly: the two sealant components were mixed correctly at 37°C and at the proper time ensuring the optimal application of active haemostatic agent at the treated liver sites.

## Discussion

Despite considerable progress in the treatment of HCC *in situ* procedures for the treatment of unresectable hepatic malignancies are associated with serious complications, including abdominal haemorrhage and bile leakage [11, 12, 15, 20–30]. Fibrin sealants have been used successfully in a variety of surgical and endoscopic settings to promote haemostasis and tissue sealing [18, 19, 25, 31]. Even if a perfect hepatic resection has been carried out postoperative bleeding and segregation of fluids can occur though the resection. By using fibrin sealant the hepatic resection area can be sealed and even the smallest microlesions can be closed. In addition, the fibrin excess can avoid early fibrinolysis. Optimal administration of the fibrin sealant after biopsy or to the ablated site is frequently difficult to achieve when the sealant is delivered via a catheter as occurs during percutaneous procedures. There is therefore an unmet medical need for a new delivery system that will ensure correct administration of the effective sealant to the surgical site. Our group designed and patented this novel dual lumen catheter system for the infusion of bi-component haemostatic agents. The objective was to optimize administration of the fibrin sealant during a range of clinical procedures. We also tested the efficacy of this dual lumen catheter in an *in vivo* porcine model of hepatic FNA and RFA.

The results of our small-scale trial show that the patented minimally invasive percutaneous procedure-kit (MIPP-Kit) confirm that the kit is significantly easier to use in that much less force is require to push the two substances out of the catheter and that it allows more precise delivery of active agents to the site required. Importantly, blockage or clogging of the catheter tip did not occur. Investigators did not report any difficulties in inserting the needle into the site of infusion probably as a result of the fact that the needle was designed with an oblique tip that facilitated easy entry.

The dual-lumen kit was also shown to be effective in that all animals survived in good conditions until the day of hepatectomy and euthanasia (except one non-treatment related death). In all pigs, bleeding was stopped within 3 minutes of the application of the fibrin sealant and no cases of recurrent bleeding recurrence were observed. FNA and RFA were not associated with any complications and blood tests did not reveal any abnormal coagulation parameters.

## Conclusions

While we acknowledge that this was a small scale study in animals, we feel confident in concluding that our patented dual lumen kit provides distinct advantages in the administration of fibrin sealant in terms of ease-of-use and improved delivery. The haemostatic treatment was effective and well tolerated and both FNA and RFA procedures could be successfully and safely performed. We look forward to the possibility of conducting larger trials with this novel system.

## Abbreviations

FNA: Fine needle aspiration
RFA: Radiofrequency ablation
HCC: Hepatocellular carcinoma
US: Ultrasound
CT: Computed tomography
MR: Magnetic resonance
NADH: Nicotinamide adenine dinucleotide
MIPP-Kit: Minimally invasive percutaneous procedure-kit.
